# Sex Dimorphism in Pulmonary Hypertension: The Role of the Sex Chromosomes

**DOI:** 10.3390/antiox10050779

**Published:** 2021-05-14

**Authors:** Daria S. Kostyunina, Paul McLoughlin

**Affiliations:** Conway Institute, School of Medicine, University College Dublin, Dublin D04 V1W8, Ireland; daria.kostyunina@ucdconnect.ie

**Keywords:** pulmonary arterial hypertension, sex chromosomes, hypoxia, BMPR2, CAV1, apoptosis, inflammation, remodelling, metabolism, non-coding RNA

## Abstract

Pulmonary hypertension (PH) is a condition characterised by an abnormal elevation of pulmonary artery pressure caused by an increased pulmonary vascular resistance, frequently leading to right ventricular failure and reduced survival. Marked sexual dimorphism is observed in patients with pulmonary arterial hypertension, a form of pulmonary hypertension with a particularly severe clinical course. The incidence in females is 2–4 times greater than in males, although the disease is less severe in females. We review the contribution of the sex chromosomes to this sex dimorphism highlighting the impact of proteins, microRNAs and long non-coding RNAs encoded on the X and Y chromosomes. These genes are centrally involved in the cellular pathways that cause increased pulmonary vascular resistance including the production of reactive oxygen species, altered metabolism, apoptosis, inflammation, vasoconstriction and vascular remodelling. The interaction with genetic mutations on autosomal genes that cause heritable pulmonary arterial hypertension such as bone morphogenetic protein 2 (BMPR2) are examined. The mechanisms that can lead to differences in the expression of genes located on the X chromosomes between females and males are also reviewed. A better understanding of the mechanisms of sex dimorphism in this disease will contribute to the development of more effective therapies for both women and men.

## 1. Introduction

Pulmonary hypertension (PH) is a condition characterised by an abnormal elevation of pulmonary artery pressure (>20 mm Hg) and increased pulmonary vascular resistance, that frequently leads to right heart (RV) failure and death [1]. Increased pulmonary vascular resistance results from vasoconstriction, thickening of the vessel walls with encroachment into and narrowing of the lumen and reduction of the vascular bed [2]. PH is classified in five groups according to similarities in aetiology, haemodynamics, abnormalities of the pulmonary vasculature and clinical characteristics. The first group, pulmonary arterial hypertension (PAH) includes heritable forms caused by specific mutations, the most common of which are loss-of-function mutations in the bone morphogenetic protein receptor type 2 (BMPR2) and includes other types of pulmonary hypertension, such as idiopathic pulmonary arterial hypertension, that share similar pulmonary vascular abnormalities [1]. Further sub-types of pulmonary hypertension include PH due to left heart disease, PH due to chronic hypoxic lung diseases and PH due to chronic thromboembolic disease (CTEPH) [1]. 

Marked sexual dimorphism is observed in patients with pulmonary arterial hypertension with the major disease registries reporting female to male ratios that are typically greater than 2:1 and range up to 4:1 [3]. The female preponderance is observed in many different subtypes of PAH including heritable PAH, idiopathic PAH and PAH associated with specific diseases such as scleroderma, congenital heart disease, haemolytic disease, schistosomiasis and anorexigens [4,5,6,7,8,9,10].

Curiously, despite being much more common in females, the prognosis in females with PAH is better than that in males [11]. This observation has been called the “oestrogen paradox” and it implies that there must be important, fundamental differences in the mechanisms that cause pulmonary hypertension in males and females or difference in factors that modulate pulmonary hypertension. Understanding these differing mechanisms is central to developing more effective treatments for patients of both sexes. The term “sex” is used here to mean biological differences between males and females that arise due to differences in the sex chromosomes, the gonads (ovaries or testes) and the endogenously produced sex hormones [12]. 

More recently, it has become clear that the sex hormones cannot completely explain the sexual dimorphism of PAH and in this review we focus on the role of the sex chromosomes in producing this dimorphism, highlighting particularly the impact of genes encoded on the sex chromosomes on oxidant pathways.

## 2. Sex Hormone Dependent and Independent Differences between Males and Females

The potential roles of oestrogens and other sex hormones in producing the sex dimorphism observed in pulmonary hypertension have been extensively investigated and it is clear that they make important contributions to the differences between males and females [11,13]. For example, right ventricular function is better preserved in female patients with PAH than in male patients [14,15,16]. Better female right ventricular function is also seen in patients with pulmonary hypertension caused by chronic hypoxic lung disease and that caused by left heart disease [17,18]. Exogenous oestrogens protect right ventricular function in mice and rats with Sugen-hypoxia induced pulmonary hypertension, and this protective effect is lost in both mice and rats following oestrogen receptor 2 knockout [14,15,16]. These data suggest that oestrogens can contribute to the longer survival in females with PAH through a beneficial action on right ventricular function. 

While oestrogen actions can account for improved right ventricular function in females with PAH, this mechanism clearly cannot account for the higher incidence of PAH in females. Additionally, there are other clinical observations that are not compatible with oestrogens as a driver of the higher incidence in females. For example, loss of ovarian oestrogen production due to early menopause leads to a subsequent increased incidence of PAH when compared to women with a menopause at a normal age [19]. In cohorts of patients diagnosed with different forms of PAH (idiopathic, caused by connective tissue disease and congenital heart disease) for the first time after the 65 years of age (i.e., at an age after normal menopause), there remains a strong preponderance of female patients [20]. Populations of prepubescent children with various forms of pulmonary hypertension have a female preponderance of approximately 1.6:1.0 [21,22]. Thus, a higher incidence of PAH is seen in females even in the absence of ovarian oestrogens. 

Data from studies in animal models of pulmonary hypertension are also difficult to reconcile with the less severe PAH observed in female patients. For example, endogenous oestrogen production increased pulmonary arterial pressure and pulmonary vascular remodelling in female mice and rats with hypoxia or Sugen-hypoxia induced pulmonary hypertension [23,24,25]. In contrast, exogenous oestradiol attenuated pulmonary vascular remodelling and reduced pulmonary artery pressure in monocrotaline- and hypoxia-induced PH in male rats and in ovariectomised female mice [26,27,28,29,30]. More recent studies using the mouse Four Core Genotypes model together with gonadectomy have shown that sex bias in PH persists even in the absence of sex hormones confirming that there are important sex hormone independent mechanisms for the differences between PAH in males and in females [31,32]. 

Finally, cell culture experiments show differences in responses between male and female cells in the absence sex hormones. This in vitro approach facilitates investigation of the acute (activational) effects of sex hormones on cells although long lasting (organisational) effects established in the cells prior to isolation might persist [33]. Male cells produce more reactive oxygen species (ROS) than female cells in culture. This pattern has been shown in isolated cells from a range of different tissues including human dermal fibroblasts, human umbilical vein endothelial cells (HUVECs), and rat vascular smooth muscle cells [34,35,36]. Regarding pulmonary cells, mouse lung endothelial cells from males showed higher basal and maximal rates of mitochondrial respiration, higher levels of ATP production and greater proton leak than female endothelial cells [32]. Given the link of these mitochondrial processes to ROS production, such differences may have important implications for ROS mediated signalling [37,38]. These differences in ROS production are important as ROS play a central role in the development of pulmonary hypertension. ROS production is increased in response to chronic hypoxia and other causes of pulmonary hypertension and promotes pulmonary vasoconstriction and pulmonary vascular remodelling [39,40,41,42,43]. Pharmacological blockers of oxidative pathways are currently undergoing preclinical and clinical trials for PH treatment [44]. 

Taken together, these results show that ovarian oestrogen production cannot be the sole mechanism that causes the different PAH phenotype seen in males and females. A further important difference between males and females is the different compliment of sex chromosomes that each receives. Recently, considerable evidence has accumulated that genes encoded on the X and Y chromosomes also contribute to the sexual dimorphism observed in pulmonary hypertension. 

## 3. The Role of the Y Chromosome in Pulmonary Hypertension

Umar and colleagues have used the Four Core Genotypes mouse model to examine the effects of the Y chromosome in the development of hypoxic pulmonary hypertension [31]. The Four Core Genotypes mouse model permits separation of the influence of the sex chromosomes from that of the sex hormones [45]. Normally the sex determining region Y (SRY) gene is located on the Y chromosome and its expression during embryonic development leads to the formation of testes. In the absence of SRY testes do not develop and the embryo is committed to the development of ovaries [45]. In the Four Core Genotypes model a small region containing the Sry gene has been deleted from the Y chromosome (Y^−^) and instead an Sry transgene has been inserted into an autosome (Sry^+^). Breeding of an XY^−^(Sry^+^) male mouse with a standard XX female mouse results in four different combinations of gonads and sex: male XY^−^(Sry^+^), male XX(Sry^+^), female XX, female XY^−^. To remove the effects of sex hormones during adulthood and thus examine the separate effects of the sex chromosomes, Umar and colleagues removed the gonads from mice bred using the Four Core Genotypes model and 30 days later exposed them to hypoxia to induce PH [31]. Mice with X and Y chromosomes had lower RV systolic pressure (RVSP) and reduced pulmonary vascular remodelling when compared to mice with two X chromosomes, regardless of gonadal sex. To check whether the less severe PH in XY mice was due to the absence of one X chromosome or due to the presence of a Y chromosome, they next used the XY* mouse model. This model is based on XY* mice that during breeding produce mice with abnormal sex chromosome compliments in a recombination process [46]. Mating of an XY* male with XX mice results in four types of mice: female XX, female X0, male XY, male XXY. XY* mice with these different combinations of sex chromosomes were gonadectomised and placed in hypoxia [31]. It was found that XXY and XY mice developed less severe pulmonary hypertension than XX and XO mice, suggesting that it was the presence of the Y chromosome that exerted the protective effect observed in these inbred strains [31]. Umar and colleagues also identified four genes located on the Y chromosome whose expression was significantly downregulated in explanted lungs from males with PAH compared to lungs without PAH: lysine demethylase 5D (KDM5D), ubiquitously transcribed tetratricopeptide repeat containing, Y-linked (UTY), ubiquitin specific peptidase 9 Y-linked (USP9Y), and zinc finger protein Y-linked (ZFY) [31]. Recent preliminary data suggest that UTY is protective in experimental PH by reduction of pro-inflammatory cytokines and protection against pulmonary artery endothelial cell death [47,48].

The Four Core Genotypes model can be used to examine the effects of genes encoded on the Y chromosome on the development of PH, with the exception of the Sry gene. To assess a potential role for SRY in protecting against PAH in males, Yan and colleagues explored a link between SRY and BMPR2 and found that SRY expression stimulated BMPR2 expression in human dermal fibroblasts and HEK293 cells [49]. This finding was of interest given that reduced BMPR2 signalling due to a heterozygous loss-of-function mutation of BMPR2, plays a causative role in 75% of patients with HPAH and mice with mutated BMPR2 develop PH similar to human PAH [50,51]. Furthermore, reduced BMPR2 expression and signalling is present in other forms of PH, in which no BMPR2 mutation is present [52,53,54]. Loss of BMPR2 function leads to mitochondrial dysfunction, increased reactive oxygen species and oxidant mediated damage in vascular cells in vivo and in cell culture [55,56,57]. Given the central role that altered ROS production plays in the development of PAH, SRY may protect against PAH in males by increasing BMPR2 expression and thus attenuating the deleterious effects of loss of function of BMPR2, although it remains to be shown if this action of SRY is found in the lung in vivo. 

Overall, there is now considerable evidence that the Y chromosome has an important role in producing the sex bias in hypoxia-induced pulmonary hypertension in mice although the mechanisms by which it acts are not fully explored. However, the contribution of genes located on the Y chromosome to sex dimorphism in humans has not yet been specifically investigated and therefore it is not clear how such genes could contribute to a reduced incidence of pulmonary hypertension in males. It is possible that when another stimulus that causes pulmonary hypertension is present (e.g., BMPR2 mutation, hypoxia etc.) the products of these genes may act to reduce pulmonary vascular resistance and thus prevent clinically significant disease in circumstances that might otherwise have caused pulmonary hypertension. However, extensive further work will be needed to provide a more complete understanding of the role of the Y chromosome in human disease.

## 4. Mechanisms of Sexual Dimorphism Related to the X Chromosome

**Figure 1:**
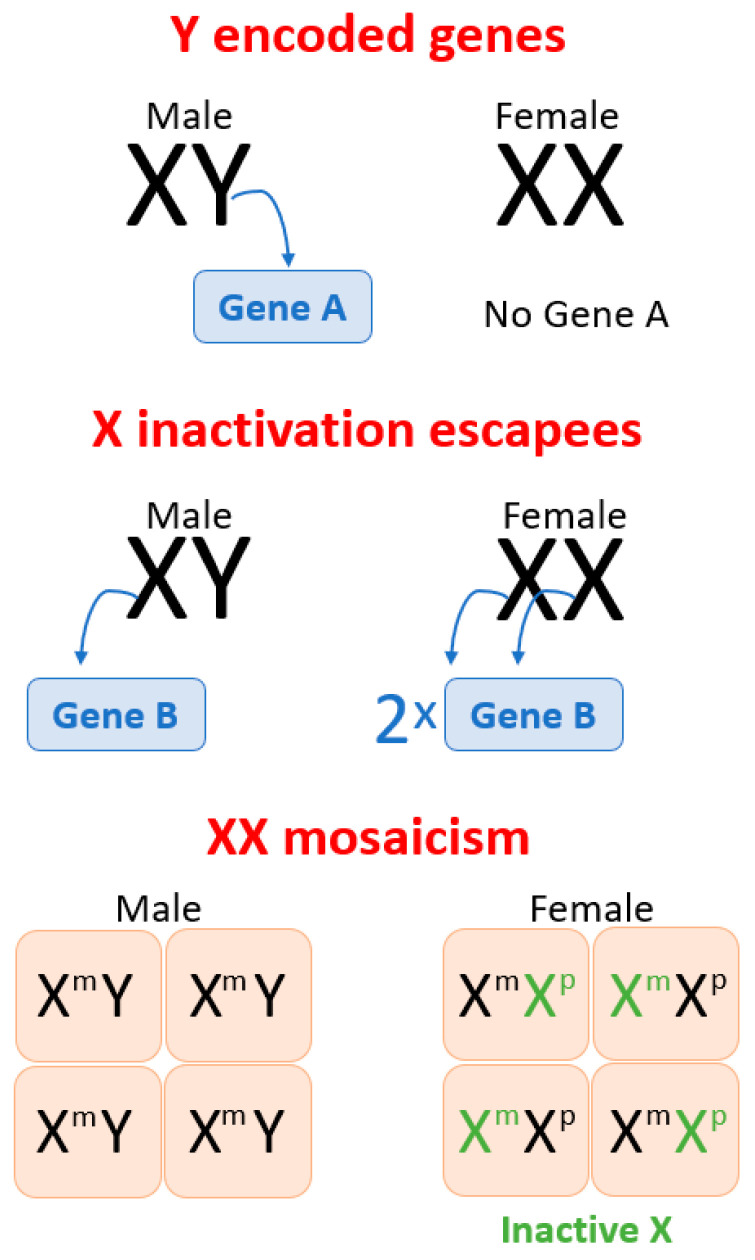
Possible mechanisms, underlying how sex chromosomes induce sex differences between males (XY) and females (XX), include: (1) genes located on the Y chromosome that are present in XY cells and not present in XX cells; (2) genes located on the X chromosome that escape X inactivation and thus are more highly expressed in female cells than in male cells; (3) XX mosaicism which is a random inactivation of one of the X chromosomes, maternal X (X^m^) or paternal X (X^p^) in different cells. This process is not completely random and, in some females, X^m^ or X^p^ is inactivated in more than 50% of cells, which leads to X skewing (not depicted on Figure 1). Males (XY) have X^m^ in every cell and hence are not XX mosaic so that mutations impairing gene function cause more severe phenotypes in males.

The X chromosome contains around 2600 genes (NCBI gene) and differences in the expression of these genes are seen in females and males due to a number of different mechanisms [58] (Figure 1). All male cells receive a single X chromosome and this is invariably a maternal chromosome (Figure 1). In contrast, all female cells have two X chromosomes, one inherited from each parent. Despite this, most genes located on the X chromosomes are equally expressed in both male and female cells because one X chromosome in each female cell is inactivated by an X encoded long non-coding RNA, X inactive specific transcript (XIST) [58]. However, 15–30% of X chromosome genes escape inactivation, which leads to a higher gene dosage in female cells [59,60,61]. Some of these escapees are consistent across all tissues while others are tissue specific. Some that were initially inactivated can reactivate in certain conditions such as aging and cancer [61,62,63]. Thus, escape from inactivation is one mechanism that can cause differing “gene doses” between male and female cells and thus cause phenotypic differences. 

The second mechanism by which sex chromosomes can lead to differences between males and females is mosaicism. In females one of the two X chromosomes in each cell is randomly inactivated [58]. Hence, in females some cells have an active maternal X chromosome while other cells have an active paternal X chromosome. Males have only one X chromosome in every cell, which is maternal in origin. Maternal and paternal X chromosomes are different because of imprinting and genetic variability [64,65]. Maternal and paternal imprinting are different and lead to differences in expression of the maternally and paternally inherited alleles of each gene. As a result of random inactivation of the X chromosome, approximately, half of female cells will have an active allele whose expression is determined by paternal imprinting while the other half will have expression determined by maternal imprinting. In contrast, in all cells in the male expression will be determined by the maternal pattern of imprinting [66]. Similarly, the maternal and paternal X chromosomes might have alleles that have different polymorphisms, which could affect gene expression or the activity of the resultant transcript.

As a result, approximately 50% of cells in a female will express one polymorphism while the other 50% will express a different polymorphism. In contrast all cells in males will express a single (maternal) polymorphism. The mosaic pattern of expression in the female provides a buffer against the effect of detrimental imprinting or a disadvantageous allele inherited from any one parent. Thus, detrimental mutations and polymorphisms in genes located on the X chromosome are usually manifested in males more profoundly than in females due to female mosaicism [58]. 

A further mechanism that can modulate the effects of mosaicism is secondary skewing [58]. In a female embryo, two populations of cells will initially develop, one expressing the paternal allele and the other expressing the maternal allele. Where one or other allele has a disadvantageous polymorphism that impairs cell growth and proliferation, the cells expressing the more advantageous allele will dominate the final population of cells in the adult organism [58]. 

## 5. The X Chromosome in Pulmonary Hypertension

We have identified 25 genes located on the X chromosome that have been implicated in the pathogenesis of PH based on evidence of altered expression and activity in clinical samples from patients with pulmonary hypertension, evidence from animal models of pulmonary hypertension or both. Some of the genes are rare X-linked variants that cause pulmonary hypertension in humans as part of complex syndromes. These genes could contribute to sexual dimorphism by one of the mechanisms outlined above and they are listed in Table 1 categorised according to their canonical functions. 

### 5.1. Reactive Oxygen Species

Interestingly, a substantial number of proteins encoded on the X chromosome are linked to pathways that alter ROS production and oxidant stress in the lung. Monoamine oxidases (MAOs) are mitochondrial enzymes involved in production of hydrogen peroxide via the catabolism of monoamines such as catecholamines and serotonin (5-HT). There are two isoforms of the enzyme MAOA and MAOB encoded on the short arm of the X chromosome at closely related loci and both are expressed in the pulmonary vasculature [67,68]. In the rat Sugen-hypoxia model of pulmonary arterial hypertension, a selective inhibitor of MAOA reduced oxidative stress in the pulmonary vasculature to normal levels and attenuated the development of pulmonary hypertension [67]. Recently, a human family has been described with Norrie disease caused by a microdeletion on the X chromosome that caused loss of norrin (NDP) but also deleted the genes encoding MAOA and MAOB [68]. Two half-brothers who inherited the microdeletion from their mother developed PAH in childhood while a further brother died in childhood following a brief illness compatible with the development of PAH. The mother was unaffected as was one female child although the kindred was too small to clearly define the consequences of the loss of the two monoamine oxidases in males [68]. It is interesting that while selective inhibition of MAOA ameliorated pulmonary hypertension, loss of both MAOA and MAOB caused the disease suggesting different functions for the two proteins in the pulmonary circulation. Elucidation of these differences will require further research. 

NADPH oxidases 1 and 2 (NOX1 and NOX2) are membrane enzymes that produce superoxide (O_2_^•−^) by transferring electron from NADPH to O_2_ and are also encoded on the X chromosome (Table 1). NOX1 protein expression is elevated in lung tissue from patients with PAH and is accompanied by an increase in ROS production and of the BMP antagonist gremlin 1 (GREM1) [71]. This was of particular interest as it has been previously shown that Grem1 plays a key role in the development of hypoxic pulmonary hypertension through its blocking action on BMP signalling [54,114]. In human pulmonary artery endothelial cells hypoxia induced NOX1, GREM1 expression and endothelial cell proliferation. NOX1 gene silencing blocked both the hypoxic induction of GREM1 and prevented the associated cell proliferation [71]. NOX1 activity promoted Grem1 upregulation by activating the transcription factor CREB. It has been previously shown that CREB is selectively activated by hypoxia in the mouse lung in vivo and is also activated in the Sugen-hypoxia model of PAH in rats [69,70,115]. 

When NOX1 expression and activity in monocrotaline-induced pulmonary hypertension in rats was inhibited, pulmonary vascular resistance was reduced and pulmonary hypertension attenuated [69]. Furthermore, hypoxia-induced pulmonary hypertension was attenuated in female NOX1^−/−^ deficient mice [72]. While all of this suggests that increased NOX1 activity cause pulmonary hypertension, it has also been reported that complete loss of NOX1 function through gene deletion in male mice (NOX^−/y^) leads to the spontaneous development of increased pulmonary vascular resistance and PH in mice in the absence of any precipitating cause [116]. This suggest different effects of NOX1 on the pulmonary vascular circulation of males and females but further research is needed to clarify this possibility. 

There is also evidence of NOX2 involvement in the pathogenesis of pulmonary hypertension. NOX2 protein is elevated in endothelial progenitor cells in hypoxia-induced PH in rats [74]. Hypoxic pulmonary hypertension is attenuated in NOX2 deleted mice together with superoxide production in the pulmonary vasculature, reduced pulmonary vascular resistance and right ventricular hypertrophy [43]. Caveolin-1 (CAV1) is a negative regulator of NOX2 in the normal pulmonary circulation and genetic ablation of CAV1 increases NOX2 expression and activity and worsens pulmonary hypertension [73]. This is of particular interest since a non-functioning mutation of CAV1 is one of the causes of heritable PAH in humans [117]. 

### 5.2. Glucose Metabolism

Glucose-6-phosphate dehydrogenase (G6PD) catalyzes the conversion of G6P to 6-phosphogluconolactone, the first step on the pentose phosphate pathway, with the formation of NADPH from NADP. The pentose phosphate pathway supplies NADPH to glutathione reductase for the regeneration of glutathione (GSH) from reduced glutathione (GSSG). This GSH is used by glutathione peroxidase for the reduction of H_2_O_2_ to H_2_O [118]. However, NADPH can also be used to support the production of superoxide radicals by NADPH oxidases, uncoupled nitric oxide synthase, and xanthine oxidase [119]. Thus, alterations in G6PD have the potential to either increase or decrease oxidant species depending on the specific context and circumstances [118]. This “Janus-like” potential may account for the seemingly conflicting roles for G6PD mutations in pulmonary hypertension. Gupte and colleagues reported that acute hypoxic pulmonary vasoconstriction depended on G6PD activity through its production of NADPH for NADPH oxidase dependent superoxide radical generation [120]. G6PD expression and activity are increased in hypoxic pulmonary hypertension [76]. Joshi and colleagues studied mice of both sexes and found that genetic deletion of G6PD in mice prevented the development of hypoxic pulmonary hypertension and furthermore that shRNA mediated knockdown of G6PD or pharmacological inhibition of G6PD partially reversed established pulmonary hypertension in the hypoxia-CYP2C44^−/−^ model of pulmonary arterial hypertension, a model in which female mice develop more severe pulmonary hypertension [78]. Interestingly, they also reported that G6PD acted to alter DNA methylation thus regulating gene expression, a novel function for G6DP contributing to the development of pulmonary hypertension. In marked contrast to those findings, Varghese and colleagues recently reported that a G6PD mutation that reduced enzymatic activity to 10% of control values caused worsening of hypoxic pulmonary hypertension and increased oxidant production in male mice [77]. The reasons for these differences may relate to the different sexes included in these studies or to other factors that will require further research to elucidate. 

### 5.3. Copper Metabolism

ATPase copper transporting alpha (ATP7A) encodes a transmembrane Cu transporter, an essential cofactor for several enzymes that catalyse electron transfer, including cytochrome c oxidase, lysyl oxidase and superoxide dismutases [121,122]. Extracellular SOD (SOD3) is a copper (Cu)-containing enzyme that is secreted and binds to the endothelial cells surface, where it scavenges extracellular superoxide radical. It colocalises with caveolin-1 together with ATP7A upon which it depends for Cu [123]. Loss of ATP7A function in mice worsens angiotensin-2 induced hypertension by reducing SOD3 activity and increasing vascular superoxide thus demonstrating an essential role for ATP7A in vascular homeostasis [124]. ATP7A is upregulated in the lung tissues and pulmonary arteries of mice with hypoxia-induced pulmonary hypertension [79]. The hypoxia mimetic cobalt chloride increases expression of ATP7A in pulmonary arterial smooth muscle cells [79]. Loss of function mutations of CAV1 with associated reduction in endothelial cell caveoli are a rare cause of inherited PAH [117]. Cav1^−/−^ mice develop pulmonary hypertension with increased oxidative stress and impaired endothelial function [125]. Loss of Cav1 leads to reduced ATP7A protein and reduced SOD3 activity [123]. Restoration of SOD3 activity either by over-expression of ATP7A or SOD3 restored normal endothelial function [123]. Taken together these suggest a role for ATP7A in maintaining normal pulmonary vascular resistance. Interestingly, there are reports of patients with mutations in ATP7A causing structural abnormalities of the pulmonary circulation and pulmonary hypertension [80,126]. 

### 5.4. Nutrient Sensor

OGT encodes O-linked *N*-acetylglucosamine transferase producing two different isoforms, nucleocytosolic and mitochondrial, that catalyse the addition of *N*-acetyl-d-glucosamine to proteins and thus alter protein function [127]. OGT deletion or knockdown causes impaired mitochondrial function and increased ROS production suggesting that OGT normally has an antihypertensive action by reducing mitochondrial ROS [128,129]. However, in the lungs of patients with PAH OGT expression is increased and accompanied by increased O-GlcNAc modification of proteins. Moreover, higher levels of OGT were associated with a poorer prognosis. Knockdown or pharmacological inhibition of OGT in pulmonary vascular smooth muscle cells isolated from patient with PAH reduced their proliferation rates to control levels suggesting a central role for this enzyme in vascular remodelling in the hypertensive lung [81]. Taken together, these data suggest that increased OGT expression and activity contributes to the development of pulmonary hypertension by altering protein function through O-GlcNAc modifications, which are distinct from its effects on ROS production. Further work is needed to identify the specific proteins involved and their actions on pulmonary vascular resistance. 

### 5.5. Apoptosis

A number of X encoded proteins regulate apoptosis which is of interest in the context of PAH where resistance to apoptosis is a prominent feature in the remodelled vasculature [130,131]. Apoptosis-inducing factor mitochondria associated-1 (AIFM1) is a mitochondrial flavoprotein that primarily regulates mitochondrial energy metabolism under normal cellular conditions and is involved in regulation of apoptosis. Loss of AIFM1 renders cells vulnerable to apoptosis and increased AIFM1 expression promotes abnormal cell proliferation [132]. A specific mutation of AIFM1, inherited in an X-linked recessive pattern, has been reported to cause pulmonary hypertension in association with neurological and skeletal abnormalities [82]. 

Histone deacetylase 6 (HDAC6) is upregulated in the remodelled pulmonary vessel of patients with PAH [83]. In contrast to most HDACs, which are located in the nucleus and regulate gene transcription by deacetylation of histones, HDAC6 is mainly localized in the cytoplasm where it regulates protein function by deacetylation. For example, it deacetylates alpha-tubulin, thus promoting cell motility [133]. In pulmonary hypertension, Boucherat and colleagues have shown that increased HDAC6 maintains the protein Ku70 in a hypoacetylated state, blocking the translocation of Bax to mitochondria and preventing apoptosis [83]. Pharmacological inhibition of HDAC6 reduced pulmonary artery pressure and vascular resistance in monocrotaline and Sugen-hypoxia rat models of PAH and mice deficient in HDAC6 showed attenuation of chronic hypoxic pulmonary hypertension [83]. 

X-linked inhibitor of apoptosis (XIAP) is another regulator of apoptosis that is encoded on the X chromosome. Its expression is elevated in persistent pulmonary hypertension of the new born (PPHN) as a result of increased mitochondrial ROS production secondary to reduction of SOD2 activity. XIAP inhibits apoptosis by binding to and inactivating caspase activity [84]. Of note, XIAP also interacts with and ubiquitinates AIFM1 at a non-canonical lysine residue. This ubiquitination does not lead to degradation of AIFM1 but reduces mitochondrial ROS production [134]. The importance of this latter action of XIAP in pulmonary hypertension requires further investigation. 

### 5.6. Inflammation

Inflammation is an important pathogenic mechanism in the development of pulmonary hypertension of differing aetiologies [135,136]. The master regulator of innate immune responses nuclear factor kappa B (NF-κB) is activated in the pulmonary circulation as a result of loss of function mutations of BMPR2 and CAV1 and also due to pulmonary hypoxia [73,137,138,139]. Inhibitor of nuclear factor kappa B kinase regulatory subunit gamma (IKBKG) encodes the regulatory subunit of the inhibitor of kappaB kinase (IKK) complex, which activates NF-κB resulting in activation of genes involved in inflammation, immunity, cell survival, and other pathways. Inactivating mutations in this gene result in incontinentia pigmenti, a multisystem disorder which is inherited in an X-linked dominant pattern and is usually lethal prenatally in males [85,86,87,140]. Female patients with the mutation can survive although with multiple abnormalities including PAH [85,86,87]. The mechanism leading to PAH in this condition is unknown at present. 

Two further genes on the X-chromosome, IL13RA1 and IL13RA2, play a role in the inflammatory processes activated in the hypertensive pulmonary circulation of some types of pulmonary hypertension. These genes encode, respectively, the low affinity interleukin 13 receptor subunit alpha 1 and the high affinity interleukin 13 receptor subunit alpha 2 [88,89,90]. In contrast, when IL-13 binds to the low-affinity IL-13Ra1, the IL-4Ra is recruited into the signaling complex, forming a high-affinity receptor which leads to tyrosine phosphorylation and downstream signalling. When IL-13 binds to IL-13 receptor subunit alpha 2, there is no signalling and this subunit is thought to act as a “decoy” receptor that reduces IL-13 signalling. IL13RA2 is upregulated in idiopathic PAH and in murine models of schistosomiasis-induced pulmonary hypertension [88,90]. IL13RA2^−/−^ mice (leading to increased IL-13 signalling) chronically infected with schistosomiasis develop worse pulmonary hypertension and pulmonary vascular remodelling whereas IL13RA1^−/−^ mice have reduced remodelling [88,90].

### 5.7. Vascular Remodelling

Pulmonary vascular remodelling and vasoconstriction are central processes in the development of pulmonary hypertension and six proteins involved in these processes are encoded on the X chromosome (Table 1). Apelin (APLN), angiotensin I converting enzyme 2 (ACE2) and angiotensin II receptor type 2 (ATGR2) cause vasodilatation and normally inhibit the development of pulmonary hypertension and in each case reductions of protein expression have been shown to contribute to the development of disease [14,91,92,93,94,95,96,97]. Vascular remodelling requires cellular proliferation and migration. Filamin A is an actin binding protein that cross-links actin filaments and links actin filaments to membrane glycoproteins. Recently it has been found to be elevated in the lungs of patients with pulmonary arterial hypertension secondary to congenital heart disease [98,99]. Interestingly, a mutation in filamin A gene (FLNA) has been identified in two sisters both of whom had pulmonary arterial hypertension. The mutation was not detected in their father suggesting that it was probably inherited from their mother in an X-linked fashion, although this could not be genetically confirmed as she was deceased [98]. Thymosin beta-4 (TMSB4X) is a G-actin sequestering protein that regulates migration and proliferation of endothelial cells [141]. Thymosin beta-4 was previously shown to be protective against monocrotaline-induced PH in mice by upregulation of Notch3 [100]. Tissue inhibitor of metallopeptidase 1 (TIMP1), an X encoded protein required for normal extracellular matrix remodelling is elevated in pulmonary blood vessels in the plasma of patients with PAH and is a predictor of poor prognosis. However, whether these increases are causative or secondary to the disease process remains to be determined [101,102,103,104]. 

### 5.8. Regulation of Transcription

Genes located on the X chromosome encode a number of transcription factors and non-coding RNAs that alter gene expression and contribute to the development of pulmonary hypertension (Table 1). Importantly, this represents a mechanism by which genes encoded on the X chromosome can alter the expression of autosomal genes in a sex specific way [62,142]. The inflammation associated with PAH leads to elevated granzyme B which cleaves intersectin-1. One of the cleavage products of intersectin1, EHITSN, substantially upregulates the expression and activity of the long non-coding RNA Xist in female pulmonary artery endothelial cells [105]. Pulmonary arterial endothelial cells from female patients with PAH demonstrate this upregulation of XIST but it is not seen in cells from males with PAH. XIST upregulation leads to increased expression of the X encoded transcription factor ELK1 triggering a proliferative response in these cells [106]. Furthermore, the upregulation of XIST represses expression of Kruppel-like factor 2 (KLF2), encoded on chromosome 19, in female pulmonary arterial endothelial cells, mimicking the repression of KLF2 observed in human pulmonary arterial hypertension [106,143]. Another example of an autosomally encoded protein whose expression is regulated by XIST is HIF-1α [144]. This may be of importance in the context of pulmonary hypertension given the known role of HIF-1α in pulmonary hypertension but to date this action of XIST has only been reported in colonic cancer cells [145,146].

Methyl-CpG binding protein 2 (MECP2) protein is encoded on the X-chromosome and binds to methylated DNA promoter regions thus altering expression of specific genes, causing repression of some (15% of genes) and activation of other (85% of genes) [147]. Duplication of this gene causes increased expression of MECP2 protein and leads to specific neurodevelopmental and immunological abnormalities collectively called the MECP2 duplication syndrome. This syndrome is much more common and more severe in males, although the severity and exact spectrum of abnormalities is variable [148]. More recently, it has been recognized that PAH is commonly found in males with MECP2 duplication syndrome and contributes to early death [107,108]. The exact mechanisms leading to pulmonary hypertension are unknown but transgenic mice overexpressing MECP2 develop very severe pulmonary infections in response to influenza virus and these infections are complicated by features of pulmonary hypertension, a response not seen in wild type control mice where influenza virus causes a much more benign infection [149]. 

### 5.9. Non-Coding RNAs

In addition to the regulation of gene effects by regulation of gene expression, gene effects are also regulated by post-transcriptional mechanisms. miRNAs regulate expression of more than 30% of human genes by translation inhibition or by targeting mRNAs for degradation [150,151]. MicroRNAs on the X-chromosome can escape X chromosome inactivation and thus be more highly expressed in female cells and produce sex specific differences in cellular behaviour [34]. Three microRNAs encoded on the X-chromosome have been shown to promote the development of pulmonary hypertension and to be upregulated in rodent models of pulmonary hypertension (Table 1). One of these, MIR223, is reduced in the lungs and serum of female patients with congenital heart disease associated PAH but unchanged in males with this condition [112]. It is downregulated in mouse and rat models of pulmonary hypertension by a HIF1α-dependent mechanism. Restoration of MIR223 activity in vivo using specific agomir or mimetic attenuated the hypoxia-induced increase in pulmonary artery pressure and distal arteriole muscularization in mice and blocked smooth muscle cell migration and proliferation [112,152]. 

Long non-coding RNAs can alter gene effects by multiple mechanisms including modulation of transcription, mRNA splicing, mRNA stability and mRNA translation [153]. While the X encoded long non-coding RNA XIST is best known for its role in X chromosome inactivation, it can also regulate the effects of autosomal genes in a sex specific manner as outlined above [106]. Interestingly, a number of the X encoded genes listed in Table 1 that participate in PH development (including MAOA, NOX2, G6PD, ATP7A, XIAP, IKBKG, TIMP1, TMSB4X, FLNA, XIST) escape inactivation in other contexts although whether this occurs in the different forms of pulmonary hypertension has not been investigated to date [60,61,154,155,156,157].

### 5.10. Potential Roles of X-Linked Genes in Sex Dimorphism

While many of the genes located on the X chromosome that we have discussed (Table 1) have been shown to be altered in human disease, in animal models of pulmonary hypertension or both, altered expression of these in isolation does not cause the disease. As shown by genetic mutation studies in animal (Table 1), complete of loss of function of most of these genes did not cause pulmonary hypertension in the absence of any other cause, but worsened hypertension initiated by well-known stimuli such as chronic hypoxia, chronic schistosomiasis infection, monocrotaline and bleomycin. This suggests that differences in expression of these genes (e.g., due to escape from X inactivation) or differences in the activity of their products (e.g., due to inheritance of different polymorphisms) between males and females could modify the course of pulmonary hypertension differently in the two sexes once the disease had been initiated by another cause. Such differences in gene expression and activity could also alter the vulnerability of males and females to other causes of pulmonary hypertension differently and thus contribute to the differences in the rate of diagnosis of pulmonary hypertension in males and females. 

A particular example of interaction between X-linked genes and other stimuli causing pulmonary hypertension is found when autosomal mutations cause pulmonary hypertension in a sex dimorphic pattern. BMPR2 mutations, the first identified and most common of the heritable mutations, lead to PAH development in approximately 20% of those who inherit a mutation suggesting that a second pro-hypertensive stimulus, a “second hit”, is required to produce disease. Furthermore, even though BMPR2 is an autosomal gene, females with the mutations more commonly develop pulmonary hypertension than males [50,51]. Two other autosomal genes have been clearly shown to produce sexual dimorphism in animal models. Deletion of Cyp2c44 in mice leads to more severe hypoxic pulmonary hypertension in female mice compared to males [78]. More recently, a loss-of-function mutation in NFU1 has been identified as another autosomal gene mutation that cause spontaneous development of pulmonary hypertension with higher penetrance in females [158,159]. In human patients this mutation causes reduced activity of respiratory chain complexes 1 and 2, elevated lactate, hyperglycaemia, complex neurological deficits and pulmonary hypertension [159]. Reproduction of this NFU1 mutation in rats by CRISPR/Cas9 engineered gene modification caused spontaneous development of pulmonary hypertension with a greater female penetrance [158]. These autosomal mutations might produce disease with sex dimorphism by interacting with genes located on the X chromosome. For example, as outlined above (Section 5.1), data from previous work show that NOX1 interacts with the BMPR2 pathway through an increase in GREM1 expression [54,70]. Separate work showed that GREM1 is a modifier gene in families with heritable PAH due to BMPR2 mutation [114]. Members of a kindreds with the BMPR2 mutations who did not develop the disease had low expression of GREM1 while members of the kindred who developed the disease had high GREM1 expression, a “second hit” that also acts to reduce BMP signalling. Further work is needed to determine whether other genes on the X chromosome interact with autosomal genes in analogous ways and whether such interactions contribute to sex dimorphism in humans. 

## 6. Conclusions

The sex chromosomes play a potentially important role in the sex dimorphism observed in pulmonary hypertension. The Y chromosome is protective in hypoxia-induced pulmonary hypertension but the specific genes and mechanisms that are responsible for this have not yet been identified. There are also a large range of candidate genes located on the X chromosome that could contribute to sex dimorphism in pulmonary hypertension. Hence, genes located on the sex chromosomes are promising targets for further research to enhance our understanding of the differing disease mechanisms in males and females and to develop more effective, sex specific therapeutic interventions.

## Figures and Tables

**Table 1:** X chromosome genes implicated in pulmonary hypertension.

Function	Gene Name	Gene	Species and PH Model/Group	References
ROS production	Monoamine oxidase A	MAOA *^&^	Rat SuHx	[67]
			Human PAH; PH	[67,68]
	Monoamine oxidase B	MAOB *	Human PH	[68]
	NADPH oxidase 1	NOX1 ^$^	Rat MCT; SuHx	[69,70]
			Human PAH	[71,72]
	NADPH oxidase 2	CYBB (NOX2) ^$^	Rat MCT; HPH	[73,74]
Glucose metabolism	Glucose-6-phosphate dehydrogenase	G6PD	Rat SuHx; HPH	[75,76]
			Mouse G6pd deficiency; Cyp2c44^−/−^	[77,78]
Copper metabolism	ATPase copper transporting α	ATP7A *^$^	Mouse HPH Human PH	[79] [80]
Nutrient sensor	O-linked *N*-acetylglucosamine (GlcNAc) transferase	OGT	Human IPAH; HPAH	[81]
Apoptosis	Apoptosis inducing factor mitochondria associated 1	AIFM1 *	Human PH	[82]
	Histone deacetylase 6	HDAC6 ^&^	Rat SuHx; MCT	[83]
			Human PAH	[83]
	X-linked inhibitor of apoptosis	XIAP	Human PPHN	[84]
Inflammation	Inhibitor of NF-kappa-B kinase regulatory subunit γ	IKBKG * (NEMO)	Human PH	[85,86,87]
	Interleukin 13 receptor subunit α 1	IL13RA1 ^$^	Mouse Shist	[88]
	Interleukin 13 receptor subunit α 2	IL13RA2 ^$^	Mouse HPH; IL-13 PH; Shist	[88,89,90]
			Rat MCT	[89]
			Human IPAH	[89]
Vascular remodelling	Apelin	APLN ^&^	Rat SuHx; HPH; MCT; PAB	[14,91]
			Human IPAH	[91,92]
	Angiotensin I converting enzyme 2	ACE2 ^$^	Mouse SuHx	[93,94]
			Rat MCT	[93]
			Human IPAH	[93,94]
	Angiotensin II receptor type 2	AGTR2 ^&^	Rat MCT; Bleo	[95,96,97]
	Filamin A	FLNA *	Human CHD-PAH; HPAH	[98,99]
	Thymosin beta 4 X-linked	TMSB4X ^&^	Mouse MCT	[100]
	TIMP metallopeptidase inhibitor 1	TIMP1	Rat MCT + Left pneumonectomy	[101]
			Human PAH-CTD; IPAH; CTEPH; PH-LHD	[102,103,104]
Regulation of transcription	ETS transcription factor ELK1	ELK1 ^#^	Mouse SuHx	[105]
			Human IPAH	[105,106]
	Methyl-CpG binding protein 2	MECP2 *^$^	Human PH	[107,108]
Non-coding RNAs	MicroRNA 98	MIR98	Mouse HPH; SuHx	[109]
			Rat HPH	[110]
			Human IPAH	[109]
	MicroRNA 223	MIR223 ^#&^	Mouse HPH	[111]
			Rat HPH; MCT	[112,113]
	MicroRNA 424	MIR424 ^&^	Human PAH; CHD-PAH	[112]
			Mouse SuHx	[91]
			Rat MCT	[91,113]
	X inactive specific transcript	XIST ^#^	Human PAH	[91]
			Human IPAH	[106]

* Rare X-linked genetic variants that cause pulmonary hypertension in humans as part of complex syndromes. ^$^ Genetic manipulation in rodent models confirms contribution of PH pathogenesis. ^&^ role in pathogenesis of PH demonstrated by replacement of gene product or use of specific antagonists (including antagomirs) # genes that have been found to demonstrate sex dimorphism in human serum or cells. SuHx, Sugen-hypoxia. MCT, monocrotaline. HPH, hypoxia-induced PH. HPAH, heritable PAH. PPHN, persistent pulmonary hypertension of the new born. Cyp2c44^−/−^, hypoxia-Cyp2c44^−/−^ mouse model. Shist, Schistosomiasis-induced PH. IL-13 PH, spontaneous PH in lung-specific IL-13-overexpressing transgenic mice. PAB, pulmonary artery banding. Bleo, bleomycin. CHD, congenital heart disease. CTD, connective tissue disease. CTEPH, chronic thromboembolic pulmonary hypertension. LHD, left heart disease.
